# Novel Wearable Electrodes Based on Conductive Chitosan Fabrics and Their Application in Smart Garments

**DOI:** 10.3390/ma11030370

**Published:** 2018-03-02

**Authors:** Haiming Qin, Junrong Li, Beihai He, Jingbo Sun, Lingrui Li, Liying Qian

**Affiliations:** 1 State Key Laboratory of Pulp and Paper Engineering, South China University of Technology, Guangzhou 510640, China; qinhm1994@163.com (H.Q.); ppebhhe@scut.edu.cn (B.H.); 2 A Mu (Shenzhen) New Technology Co., Ltd., Shenzhen 518109, China; jbsun123@163.com (J.S.); lingruili70@163.com (L.L.)

**Keywords:** wearable electrodes, chitosan, electroless plating, silver nanoparticles, smart garments

## Abstract

Smart garments, which can capture electrocardiogram signals at any time or location, can alert others to the risk of heart attacks and prevent sudden cardiac death when people are sleeping, walking, or running. Novel wearable electrodes for smart garments based on conductive chitosan fabrics were fabricated by electroless plating of silver nanoparticles onto the surfaces of the fibers. The electrical resistance, which is related to the silver content of the composite fabrics, can be as low as 0.0332 ± 0.0041 Ω/sq due to the strong reactivity between amine groups and silver ions. After washing these fabrics eight times, the electrical resistance remained below 1 Ω/sq. The conductive chitosan fabrics were applied to smart garments as wearable electrodes to capture electrocardiogram signals of the human body in static state, jogging state, and running state, which showed good data acquisition ability and sensitivity.

## 1. Introduction

The electrocardiogram (ECG) is a simple and non-invasive test for recording the electrical activity of the heart. The ECG signal has three main waves called P, QRS, and T, which represent atrial depolarization, ventricular depolarization, and ventricular repolarization, respectively [1]. The information in the form of the ECG signal is useful in detecting cardiovascular diseases. An ECG monitoring system that is available at any time and location is very important to people with cardiac problems. It has been reported that estimates of the incidence of sudden cardiac death in marathons were in the range of 0.6–1.9 per 100,000 runners [2]. Sleep in itself is associated with changes in autonomic activity and can be considered an autonomic stressor for the heart [3]. Sleep apnea is the main reason of sudden cardiac death at night. A large number of outpatients with severe mental illness are at high risk of obstructive sleep apnea [4,5]. Sudden infant death syndrome is also a main reason for sleep-related infant deaths [6,7], while cardiac arrhythmias can be a cause of sudden infant death, especially in families that are suffering from any cardiac disease [8]. Sudden cardiac death is also the most frequent cause of death during mountain activities, such as hiking at high altitudes, especially for people with underlying coronary heart disease [9]. It is very important to detect and diagnose fatal arrhythmias accurately as early as possible and take effective measures quickly. Smart garments with ECG acquisition devices are becoming promising solutions to the accurate prediction of sudden cardiac death. ECG electrodes, which are special electrodes that act as the interface between the human body and electronic products, are responsible for collecting biosignals, and they should be highly conductive without skin irritation. Conductive textile is one of the most important ECG electrodes [10,11,12] and a more practicable emerging strategy for preparing conductive textile fibers is to coat insulating fibers with conductive thin layers, such as nanosilver, to maximize electrical conductivity (6.3 × 10^−7^ S/m) among the miscellaneous metals. The challenge of creating coated conductive fibers involves the delaminating of the metal layer from the fiber surface that is caused by repeated abrasion and wash, which results in serious conductivity loss during use. Conductive composite fibers have been developed by melt or wet spinning using conductive fillers, such as metal particles, carbon nanotube (CNT), or grapheme [13,14]. Electrical conductivities of the composite fibers are generally low since the loading level of the conducting filler is limited for spinnability [15]. Another challenge in the practical applications of conductive composite fibers is the maintenance of high conductivity over a high strain region [16]. It is almost impossible to fabricate conductive polymers into flexible fibers with high electrical conductivity.

Electroless plating is a simple and cost-effective method to coat a nanosilver layer on fibers in order to obtain conductive textiles with high conductivity. However, conductivity deteriorates significantly with washing [17,18] due to the low affinity between nanosilver and fiber surface. Nanosilver-coated fabrics of some synthetic and natural fibers that displayed various electrical resistances, such as 4.5 Ω/sq for nylon [19], 1.3 Ω/sq for polyester [17], 0.38 Ω/cm for aramid [20], and 37.0 ± 1.8 Ω for cotton [21], respectively. Some functional groups, such as hydroxyl, carboxyl, amine, and thiol, exhibited strong interactions with silver ions, and consequently, improved the conductivity and stability of the conductive fabrics. The cotton fabric was modified to introduce thiol groups and was coated with nanosilver with an electric resistance of 3.92 ± 0.18 Ω [22]. Chitosan, which is a polysaccharide consisting of a large number of hydroxyls and amine groups, is a promising candidate for fabricating the wearable electrodes for ECG acquisition devices because the amine groups are strongly reactive with metal ions due to its nitrogen atoms holding free electron doublets [23]. Furthermore, chitosan textiles exhibit antibacterial activity and are comfortable, which is very important in smart garments. 

In this paper, novel wearable electrodes were fabricated by electroless plating of silver nanoparticles onto the surfaces of the chitosan fabrics. The relationship between electrical resistance and the silver content of the composite fabrics as well as the washability of the conductive textile were investigated. The performance of the obtained chitosan fabrics for ECG acquisition as wearable electrodes was evaluated in different motions of the human body.

## 2. Materials and Methods

### 2.1. Materials

Non-woven chitosan fabrics were received from Hismer Bio-technology Co., Ltd., Tai’an, China. Silver nitrate (AgNO_3_, >99.8%) was purchased from Sinopharm Chemical Reagent Co., Ltd., Shanghai, China. Sodium hydroxide (NaOH), aqueous ammonia (25 wt %), and ethanol were obtained from Tianjin Fuyu Fine Chemical Co., Ltd., Tianjin, China. Glucose was purchased from Tianjin Fu Chen Chemical Reagent Factory. All of the reagents were of analytical grades and used as received. Laundry detergent was obtained from Guangzhou Liby Enterprise Group, Guangzhou, China. The AMSU ECG-shirt was developed by A Mu (Shenzhen) New Technology Co., Ltd., Shenzhen, China.

### 2.2. Preparation of Conductive Chitosan Fabrics

Chitosan fabrics were first cleaned three times in ethanol and deionized water alternately, before being activated in 20 g/L NaOH solution. Chitosan fabric (5 cm × 5 cm) was immersed in the silver ammonia solution, which was prepared by the addition of 300 µL NaOH solution (1 N) and 2.4 mL ammonia (25 wt %) to 60 mL of AgNO_3_ solution for 3 min. The reducing agent solution composed of dissolved glucose in mixture of 60 mL distilled water, before 6 mL ethanol was added to reduce silver ions into silver nanoparticles for 5 h at 45 °C. The mass ratio of AgNO_3_ to glucose was 1:1. The treated fabrics were rinsed with distilled water and dried at 60 °C for 2 h to obtain the silver-plated chitosan fabrics. Three repeated samples were obtained for further characterizations. 

### 2.3. Characterization of Conductive Fabrics

Scanning electron microscope (EVO 18, ZEISS, Oberkochen, Germany) was applied to characterize the surface morphologies of the fibers. The silver content of the fabrics was calculated according to the following equation:

(1) ω = (m_1_ − m_0_)/m_0_,

where m_0_ and m_1_ are the mass of pristine fabrics and silver-plated fabrics, respectively. The weight of fabrics and silver content were obtained using the average value from three repeats. The electrical resistance of the silver-plated fabrics was measured via the four-probe tester (KDB-1, Kunde Science Co., Guangzhou, China), with ten measurements obtained for each sample and the electrical resistance being the average of thirty measurements.

### 2.4. Wash Testing

To investigate washability, the fabrics that were made from 5 g/L and 7.5 g/L AgNO_3_ concentrations were immersed in 4 g/L commercial non-ionic detergent at the oscillation rate of 350 rpm in 40 °C for 30 min for a single washing. 

### 2.5. Capture of ECG Signals

The Ag-plated fabrics were applied as wearable electrodes to capture ECG signals. The dry electrode was glued by hot melt glue on the inside of the sportswear. The V1 lead position (4th intercostal space, right of sternum) and V2 lead position (4th intercostal space left of sternum) were selected in the experiment. There was a host (AMUSU V6-L dynamic electrocardiogram monitor, A Mu, Shenzhen, China) outside the sportswear. The conductive chitosan fabrics were connected to the host by wires. The electrical signals that were produced by the heart were captured by the electrodes in real time and transmitted to the host. The electrical signal was processed by the host through the algorithm and converted into the corresponding ECG data. The data was transmitted to the mobile phone APP (bodylistener, 1.1.3, A Mu, Shenzhen, China) via Bluetooth 4.0. The host ECG data were analyzed by the APP and dynamic electrocardiogram was drawn by the APP. The obtained smart garment was used on to acquire ECG waveform of a person in static (0 km/h) and dynamic (3 km/h and 12 km/h) states for three minutes individually. The signal was filtered before being plotted via a FIR (finite impulse response) filter. This study was adhered to the Declaration of Helsinki. One healthy volunteer (25-year-old, male) agreed to take up the ECG acquisition experiment using the self-developed electrodes after he was informed about the nature of the study and briefed on the recording protocol. In this work, the conductive chitosan fabrics were used as dry electrodes, and no skin preparation was needed in the experiment.

## 3. Results and Discussion

### 3.1. Morphology of the Conductive Chitosan Fabrics

**Figure 1:**
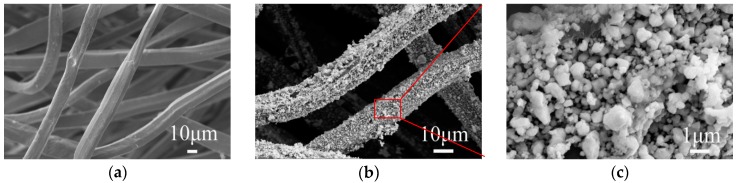
Scanning electron microscope images of pristine (**a**) and silver-plated (**b**,**c**) chitosan fibers (5 g/L AgNO_3_).

Thiol groups are normal functional groups that are introduced into cotton [22] or further produced by the reduction of silk [24], which are used to fabricate the conductive textile in a tedious procedure. However, chitosan with a high content of amine and hydroxyl groups shows good sorption capacity for transition metals, such as copper and silver ions. Ag can be easily bound to modified chitosan through the free amine and/or the vicinal hydroxyl groups [25], before being chelated and carried on the surface of chitosan fibers [26]. Amine groups of chitosan provide active sites for the formation of complexes with metallic ions [27], because a lone pair of electrons in the nitrogen atom was donated to the shared bond between the N atom and metals, which results in a decrease in the electron cloud density of the nitrogen atom and an increase in binding energy [28]. In this work, chitosan fabrics with pre-treatment were used to prepare the conductive textile. The morphologies of pristine and Ag-plated chitosan fibers were compared in Figure 1. The surface of the pristine chitosan fiber is smooth and clean; the fibers with a diameter of 15 microns were aligned disorderly in the non-woven fabrics (Figure 1a). After electroless plating, a compact and uniform silver layer is formed on the fiber surface with high coverage, although there are some loose spots due to excess silver particles being deposited on them (Figure 1b). It is obvious that the silver particles are spherical or polygon-like in shape (Figure 1c), while the size of some large particles is about 1 μm due to the agglomeration of silver nanoparticles.

### 3.2. Conductivity and Washability

The effects of AgNO_3_ concentration on the silver content and the electrical resistance are listed in Table 1. Generally, a higher AgNO_3_ concentration resulted in more silver particles being deposited on the chitosan fabrics, which resulted in lower electrical resistance. The electrical resistances of all the samples were below 0.150 Ω/sq and the lowest electrical resistance was 0.0332 ± 0.0041 Ω/sq when the concentration of AgNO_3_ was 15 g/L. The electrical resistances of the conductive chitosan fabrics are much lower than the reported results of cotton and silk [21,23,29]. Amine groups in chitosan molecules exhibited strong interactions with silver ions due to its nitrogen atoms holding free electron doublets [23] and thus, more silver ions were absorbed onto the fiber surface and the conductivity of the chitosan fabrics consequently improved. The silver content was tripled and electrical resistance decreased significantly to half when the AgNO_3_ concentrations increased from 5 g/L to 7.5 g/L. Further increases in AgNO_3_ concentrations increased both silver content and conductivity of chitosan fabrics slightly, which means that the silver content has little contribution to the electrical conductivity when the fibers were completely covered with silver. Moreover, the increased silver content resulted in a higher fabric weight and reduced softness of non-woven fabrics. Therefore, the concentration of AgNO_3_ should not be excessively high when making the electroless plating of chitosan fabrics to minimize costs. For practicality, the fabrics obtained at 5 g/L and 7.5 g/L AgNO_3_ concentrations were applied to investigate the washability in this work.

**Figure 2:**
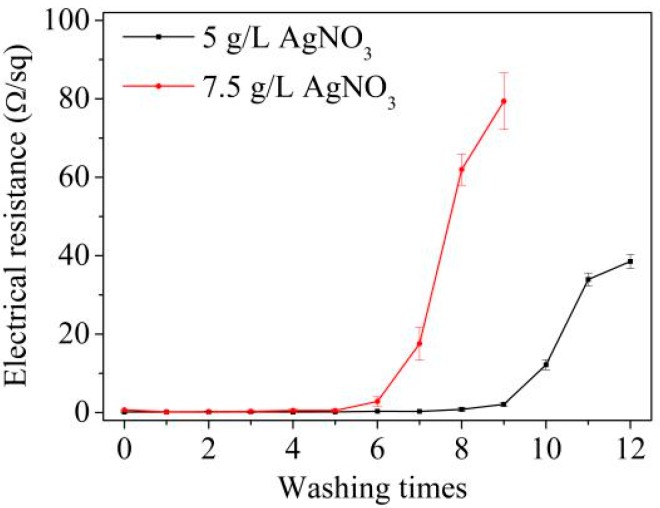
Washing fastness of the electrically conductive fabrics.

The affinity and stability of Ag nanoparticles on the surface of the chitosan fiber were investigated through the electrical resistance tests after washings in the presence of a non-ionic detergent in Figure 2. Although the electrical resistance of Ag-plated fabrics before washing was much lower for the AgNO_3_ concentration of 7.5 g/L when compared to that of 5.0 g/L, the washed garments deteriorated faster because the electrical resistance of the fabrics with the AgNO_3_ concentration of 7.5 g/L increased to 2.83 ± 1.23 Ω/sq after washing the fabric 6 times, while it reached 2.10 ± 0.46 Ω/sq after washing the fabric nine times for the fabrics with the AgNO_3_ concentration of 5.0 g/L. This might be induced by the easier exfoliation for the thick silver layer plated on the surface of chitosan fabrics. Therefore, the silver content of the fabrics should be maintained as low as possible if the electrical resistance is sufficient.

### 3.3. Application in Smart Garment

**Figure 3:**
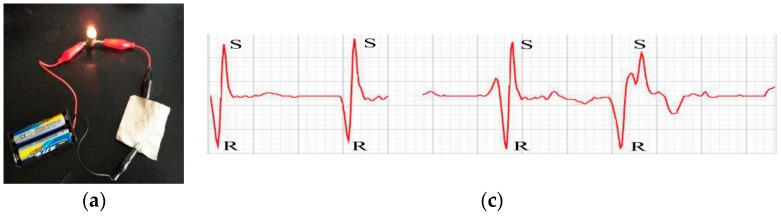

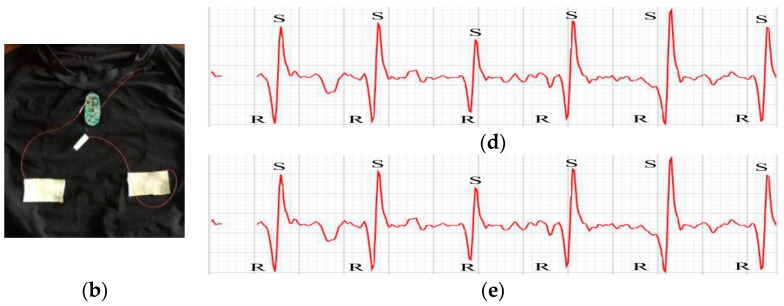
Application of conductive chitosan fabrics in smart garment (**a**): circuit diagram; (**b**): the inside of the garment; (**c**): static state (0 km/h); (**d**): jogging state (3 km/h); (**e**): running state (12 km/h).

Figure 3a showed that the Ag-plated fabrics were applied to turn on a 3 V light bulb. Two pieces of the conductive fabrics (4 cm × 7 cm) were attached as wearable electrodes to the inside of the garment (AMSU ECG-shirt) (Figure 3b) via a polyurethane hot melt adhesive by hot pressing at 150 °C for 15 s. The size of the electrodes in this work is comparable to that of other textile electrodes presented so far (e.g., 20 cm^2^ in reference [30] and 24 cm^2^ in reference [31]), although some works have presented smaller sizes (e.g., 9 cm^2^ in reference [32] and 10 cm^2^ in reference [33]). Reducing the conductive area will subsequently lead to a decrease in the quality of the recorded signal (attenuation, sensitivity to common mode noise, etc.) [34], while a smaller size caused instability in the ECG signal in this work. The garment was used to capture ECG signals from human motions at various moving states. ECG signals show P-QRS-T wave in which P wave is the first positive deflection; Q wave is any negative deflection that precedes an R wave; R wave is the first upward deflection after the P wave and it represents early ventricular depolarization; S wave is the downward deflection after the R wave; and, T wave is the positive deflection after each QRS complex and it represents ventricular repolarization [35]. Figure 3c–e showed the ECG signals for a healthy person in static, jogging, and running state, respectively. There are some gaps in the ECG signals due to the screenshot in the active capture and displaying process. When a person was in static state, the ECGs (Figure 3c) are not so steady, but the QRS complex was still recognizable. The ECGs acquired from jogging state (Figure 3d) had stable QRS-wave amplitudes with some noise. For the running state, the ECG signals (Figure 3d) showed severe fluctuations and were more contaminated by noise. Some P-waves and T-waves were not obtained. The ECG waveform can be influenced by number and position of electrodes, lead placement, gender and so on [36,37,38]. Two electrode positions (V1 and V2) were selected in the experiment and the individual person might show different ECGs. After comparing ECG signals in three states, all of the acquired signals are acceptable because all the R-peaks were detected and the obtained ECG signals can be used to calculate the heart rate [38,39]. The ECG signals acquired from the running state are better than the signals acquired from static state and jogging state.

## 4. Conclusions

Novel wearable electrodes for smart garments were fabricated by in situ reduction of silver ions into silver nanoparticles onto the surfaces of chitosan fabrics. Due to the reactivity between amine groups of chitosan and silver ions, the conductivity of the Ag-plated chitosan fabrics can be as low as 0.0332 ± 0.0041 Ω/sq and remained below 1 Ω/sq after washing the fabrics eight times. The conductive chitosan fabrics embedded in the smart garment as wearable electrodes had good capability in capturing ECG signals of the human body in various motions.

## Figures and Tables

**Table 1:** Effects of AgNO_3_ concentration on the silver content and electrical resistance.

AgNO_3_ (g/L)	5	7.5	10	12.5	15
Weight of Ag-plated fabrics(g)	0.31 ± 0.01	0.38 ± 0.03	0.44 ± 0.06	0.52 ± 0.03	0.61 ± 0.05
Silver content (g/g)	0.30 ± 0.01	1.01 ± 0.02	1.22 ± 0.05	1.48 ± 0.05	2.08 ± 0.08
Electrical resistance (Ω/sq)	0.1499 ± 0.0485	0.0700 ± 0.0103	0.0565 ± 0.0115	0.0443 ± 0.0051	0.0332 ± 0.0041
